# Comparative Metabolomic Analysis of Rapeseeds from Three Countries

**DOI:** 10.3390/metabo9080161

**Published:** 2019-08-01

**Authors:** Ruinan Yang, Ligang Deng, Liangxiao Zhang, Xiaofeng Yue, Jin Mao, Fei Ma, Xiupin Wang, Qi Zhang, Wen Zhang, Peiwu Li

**Affiliations:** 1 Oil Crops Research Institute, Chinese Academy of Agricultural Sciences, Wuhan 430062, China; 2 Key Laboratory of Biology and Genetic Improvement of Oil Crops, Ministry of Agriculture and Rural Affairs, Wuhan 430062, China; 3 Institute of Agricultural Quality Standards and Testing Technology Research, Shandong Academy of Agricultural Sciences, Jinan 250100, China; 4 Laboratory of Quality and Safety Risk Assessment for Oilseed Products (Wuhan), Ministry of Agriculture and Rural Affairs, Wuhan 430062, China; 5 Quality Inspection and Test Center for Oilseed Products, Ministry of Agriculture and Rural Affairs, Wuhan 430062, China; 6 Key Laboratory of Detection for Mycotoxins, Ministry of Agriculture and Rural Affairs, Wuhan 430062, China

**Keywords:** rapeseed, China, Canada, Mongolia, UPLC-Q-TOF/MS, metabolomics

## Abstract

Rapeseed is an important oilseed with proper fatty acid composition and abundant bioactive components. Canada and China are the two major rapeseed-producing countries all over the world. Meanwhile, Canada and Mongolia are major importers of rapeseed due to the great demand for rapeseed in China. To investigate the metabolites in rapeseeds from three countries, ultra-performance liquid chromatography-quadrupole time-of-flight mass spectrometry (UPLC-Q-TOF/MS)-based metabolomics was employed to analyze rapeseeds from China, Canada, and Mongolia. As results, 67, 53, and 68 metabolites showed significant differences between Chinese and Canadian, Chinese and Mongolian, and Canadian and Mongolian rapeseeds, respectively. Differential metabolites were mainly distributed in the metabolic pathways including phenylpropanoid biosynthesis, flavone and flavonol biosynthesis, and ubiquinone and other terpenoid-quinone biosynthesis. Among the differential metabolites, contents of sinapate and sinapine were higher in Chinese rapeseeds, while the contents of brassicasterol, stigmasterol, and campestanol were higher in Canadian rapeseeds. These findings might provide insight into the metabolic characteristics of rapeseeds from three countries to guide processing and consumption of the products of rapeseed.

## 1. Introduction

Rapeseed (*Brassica napus; Cruciferae*) is an important oil crop in agriculture worldwide, and ranks the third largest source of vegetable oil all over the world [1,2,3]. Traditional rapeseed oil contains more than 40% erucic acid [4], which could pose a risk to human health when excessive amounts are consumed [5]. In addition, traditional rapeseed also has a higher concentration of glucosinolates, and their breakdown in products could induce iodine deficiency and are fatal to pigs [6]. High erucic and glucosinolate levels therefore limit the use of rapeseed. With the development of breeding methods and production technology, rapeseed contains lower erucic acid and lower glucosinolate [5,7]. Rapeseed is widely planted in China, Canada, India, Northern Europe, and Australia based on its growth habit [8]. China and Canada are the two major rapeseed production areas and rapeseed producers. In 2016, the cultivation area of China and Canada was 7.61 and 7.99 million hectares, respectively, accounting for about 38% of the total area. The production of the two countries was 15.3 and 18.4 million tons, respectively, contributing 40% of the total production (FAOSTAT, 2018). In China, rapeseed oil serves as the main vegetable oil, particularly for the people living in the Yangtze River valley [9]. With the extraordinary economic development and rapid population growth, the demand for high-quality rapeseed oil continues to increase each year. Accordingly, as the second largest rapeseed producer, China still needs to import millions of tons rapeseeds and rapeseed oils from Canada and Mongolia, of which about 93% of rapeseeds were imported from Canada. The amount of rapeseed imported by China from Mongolia also keeps increasing year-by-year.

One of the driving forces behind the popularity of rapeseed oil is its nutritional benefits. Previous studies indicate that rapeseed oil imparts positive effects on human health. It can reduce the plasma cholesterol levels, improve insulin sensitivity, and prevent and manage ischemic stroke [4,10]. Accordingly, rapeseed oil is supposed to be one of the most effective health-promoting vegetable oils. Rapeseed oil has a lower concentration of saturated fatty acids (5–10%), higher content of monounsaturated fatty acids (44–75%), and moderate content of α-linolenic acid (9–13%). More importantly, the ratio of n-6 to n-3 fatty acids in rapeseed oil is about 2:1, which is beneficial for human health [11,12,13]. Except for fatty acids, rapeseed oil contains various micronutrients such as phytosterols, tocopherols, polyphenols, and flavonoids, which draw more and more attention from consumers and nutritionists [14]. 

Metabolomics is an approach to study small molecule metabolites in specific organs and has been widely used for qualitative and quantitative analyses [15,16]. Metabolomics was used to investigate the chemical composition of different organs of *Brassica napus L.* using UPLC-QTOF-PDAP-MS [17]. The composition of oilseed rape and turnip rape seeds has been investigated by nuclear magnetic resonance (NMR) [18] and the cell wall phenolics by UPLC-MS/MS [1]. Comparative metabolomics was employed to evaluate Chinese and North American rice [16], analyze carrots from different agronomic environments [19], distinguish *Zingiber officinale* Roscoe from two geographical origins [20], expound the profile of fruits from Tanzania [15], and characterize the metabolic changes of cultivated and wild soybean under salt stress [21].

*Brassica napus L.* is a globally important oil crop, particularly in China, which is the second largest rapeseed producer. China is also a premier customer and importer of rapeseeds. Millions of tons of rapeseeds from Canada and Mongolia are imported to China to meet the increasing demand for rapeseed. Double low rapeseed imported from Canada is well known for its quality of low contents of erucic acid and glucosinolates. In China, double low rapeseed accounts for about 80% of total rapeseeds [9]. Compared with them, the content of erucic acid in the rapeseeds imported from Mongolia are relatively higher. Apart from fatty acid composition, secondary metabolites of rapeseed from these countries are becoming of more and more concern due to their potential benefits to human beings. In the present study, UPLC-Q/TOF-MS-based comparative metabolomics was employed to investigate the chemical compositions of Chinese, Canadian, and Mongolian rapeseeds. Significantly differential metabolites between Chinese and Canadian rapeseeds, Chinese and Mongolian rapeseeds, and Canadian and Mongolian rapeseeds were identified by orthogonal partial least squares discriminant analysis (OPLS-DA) and univariate analysis, respectively. The results might provide basic information to guide oil processing to produce high-quality rapeseed oils with high nutrition values.

## 2. Materials and Methods

### 2.1. Samples and Materials

Thirty-three rapeseed samples were collected from the main rapeseed-producing areas of China in 2016 after harvest, including Zhejiang province (3), Jiangsu province (3), Anhui province (2), Jiangxi province (6), Hubei province (8), Henan province (2), Hunan province (2), Sichuan province (3), Guizhou province (2), and Yunan province (2). Seven Canadian rapeseed samples were obtained from Zhanjiang and Shenzhen customs in 2016. The samples were collected according to Chinese standard SN/T 0800.1-2016 (Inspection of cereals, oils and feedstuffs for import and export-methods of sampling and preparation of samples). Nineteen Mongolian rapeseed samples were obtained from Erenhot customs in January and February 2016. The samples were all stored at 4 °C before analysis.

HPLC-grade methanol, acetonitrile, and acetic were purchased from Fisher (Fair Lawn, New Jersey, United States). Distilled water was prepared using a Milli-Q ultra-purification system (Millipore, Bedford, MA, USA). The internal standard lidocaine was purchased from BioBioPha Company (Kunming, Yunnan, China).

### 2.2. Sample Preparation

Sample preparation was performed as described elsewhere [22]. Briefly, rapeseed samples were homogenized using a MixerMill MM400 system (Retsch Technology, Haan, Germany). Approximately 100 mg rapeseeds were dissolved in 1 mL of 70% methanol containing 0.1 mg/L lidocaine and extracted overnight at 4 °C. During the extraction process, the samples were shaken using a vortex oscillator every 10 min to ensure complete extraction. Then, the mixtures were separated by centrifugation at 10,000× *g* for 10 min. The supernatants were filtered through a microporous membrane (0.22 μm pore size) and stored in a sample vial prior to UPLC-Q-TOF/MS analysis. The blank sample was prepared as the same as the test samples, expect that no samples were added at the beginning and were injected randomly throughout the whole analysis to monitor the presence of residues. The quality control (QC) sample was made by mixing all test samples. The QC sample was analyzed at the beginning, end, and throughout the whole analysis to evaluate the stability of the analysis process and injected after every five test samples during the sample analysis.

### 2.3. UPLC-Q-TOF/MS Analysis

UPLC-Q-TOF/MS analysis was performed on an UPLC system (1290 Infinity, Aligent, Santa Clara, CA, USA) coupled with a Q-TOF/MS (6520 Q-TOF, Agilent, Santa Clara, CA, USA). The chromatographic column ACQUITY UPLC HSS T3 C18 (100 mm × 2.1 mm × 1.8 μm) was obtained from Waters, and the column temperature was kept at 40 °C during the analysis. The mobile phase A was 0.1% formic acid in water, and mobile phase B was 0.1% formic acid in acetonitrile. The flow rate was 0.4 mL/min, and the gradient program was as follows: Solvent B was increased from 5% to 95% in 11 min, held at 95% B for 1 min, then phase B was decreased to 5% within 0.1 min, and maintained for 3 min. The injection volume was 5 μL. MS data were acquired from the Q-TOF/MS equipped with an ESI ion source in positive ionization mode. The MS parameters were as follows: Nebulizer pressure at 40 psi, desolvation gas flow rate of 10 L/min at 350 °C, capillary voltage of 3.5 kV, fragment voltage of 135 V, and RF voltage of 750 V. The mass data were collected within the range of m/z 50–1000 at a scan rate of 2 spectra/s. The scan time was 500 ms/spectrum. 

### 2.4. Qualitative and Quantitative Analysis of Metabolites

The raw data obtained from LC-MS were converted to mzData format, and peak finding, filtering, and alignment were performed using XCMS. The metabolites were putatively identified by matching features in publicly available databases such as the METLIN database (http://metlin.scripps.edu/index.php), MassBank (http://www.massbank.jp), and KNAPSAck (http://kanaya.naist.jp/KNApSAcK). The tests for one sample were done in triplicate and the result of quantitative analysis of metabolite was expressed as peak area which was the average value of three independent tests.

### 2.5. Data Processing and Statistical Analysis of Metabolites

Data processing and statistical analysis of metabolites were performed on MetaboAnalyst 4.0 platform [23].

Data processing of data normalization was conducted as described elsewhere, with a little modification [20]. Pareto scaling was used instead of auto scaling in data normalization, because it can reduce the impact of noise and artifacts to improve the predictive ability of the model [24]. Principal component analysis (PCA) and OPLS-DA were used to explore the global metabolomic data. Univariate analysis method and S-plot obtained from OPLS-DA were employed to identify the different components among the three countries. The metabolites were putatively identified as differential metabolites when they met the criteria listed as follows: (a) The absolute value of reliability correlation [*p*(corr)] obtained from OPLS-DA loadings S-plot is greater than 0.8; (b) *p*-value calculated using *t*-test is less than 0.001; (c) fold change (FC) is more than twice or less than half.

Kyoto Encyclopedia of Genes and Genomes (KEGG) annotation and metabolic pathway analysis of differential metabolites were performed on MetaboAnalyst 4.0 platform [23]. KEGG metabolic pathway database, overrepresentation analysis, and pathway topology analysis were used to analyze the metabolic pathways of the differential metabolites.

## 3. Results and Discussion

### 3.1. Metabolic Profiles of Rapeseeds Collected from China, Canada, and Mongolia

Metabolomics is commonly used to study the total metabolites of a given plant tissue [11,25]. In this study, UPLC-Q-TOF/MS was employed to investigate the metabolic profiles of rapeseeds from three countries. Based on mass spectra obtained from UPLC-Q-TOF/MS, qualitative analysis was conducted by matching to spectra in the public database. As results, 152 metabolites were putatively identified in the rapeseeds collected from three countries (Table S1). The metabolites were divided into 11 groups, including alkaloids, amino acids, amino acid-related compounds, fatty acids, flavonoids, lipids, organic acids, phenylpropanoids, polyketides, terpenoids, and others. Among the metabolites, the flavonoids were the major metabolites. Phytochemical composition of rape, including the roots, stems, leaves, inflorescence, and seeds, has been identified via UPLC-Q-TOF/MS [17]. Identified compounds were assigned belonging to different metabolite classes, such as cinnamic acid derivatives, flavonoids, sinapoyl cholines, and fatty acid derivatives. Compared with this reference, quercetin 3-sophorotrioside, kaempferol 3-O-beta-D-glucosyl-(1->2)-beta-D-glucoside, rutin, quercetin 3-O-glucoside, kaempferol, isorhamnetin, ferulate, sinapate, sinapine, 1-O-sinapoyl-beta-D-glucose, (9Z,12Z)-(8R)-hydroxyoctadeca-9,12-dienoic acid, (6Z,9Z,12Z)-octadecatrienoic acid, linoleic acid, and octadecenoic acid were also putatively identified in this study and classified as flavonoids, phenylpropanoids, and fatty acids.

### 3.2. PCA of Rapeseeds from Three Countries

**Figure 1:**
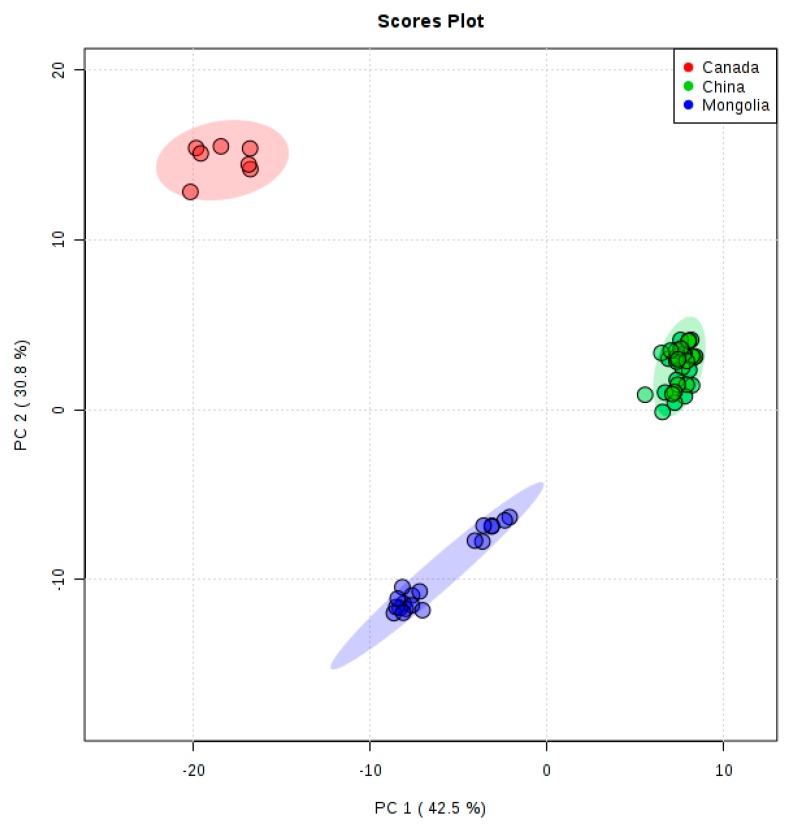
Principal component analysis (PCA) scores plot for rapeseed collected from China (green), Canada (red), and Mongolia (blue). The distinct separation of Chinese, Canadian, and Mongolian rapeseeds indicates the presence of metabolites with significant difference.

PCA is an unsupervised method, which plays an important role in the grouping and discrimination of chemicals in food and medicines [21]. PCA was performed on the metabolites of the rapeseed samples collected from China, Canada, and Mongolia to explore the global metabolomic data. The PCA results are shown in Figure 1 and all the rapeseed samples were within 95% confidence intervals. Chinese, Canadian, and Mongolian rapeseeds were clearly divided into three classes. PC1 and PC2 could explain 42.5% and 30.8% of the total variance of the data, respectively. The obvious discrimination of the three classes meant that the metabolic profiles of rapeseeds from the three countries were significantly different.

### 3.3. OPLS-DA of Rapeseeds Collected from Three Countries

OPLS-DA is a rotated and powerful supervised modeling method used for the identification of variables that drive group separation. The S-plot obtained from OPLS-DA could visualize the variable influence in the model and identify statistically significant and potentially biochemically significant metabolites [24]. To identify the different metabolites between the Chinese and Canadian rapeseeds, Chinese and Mongolian rapeseeds, and Canadian and Mongolian rapeseeds, OPLS-DA models and S-plots were established, respectively.

OPLS-DA score plots for Chinese and Canadian rapeseeds, Chinese and Mongolian rapeseeds, Canadian and Mongolian rapeseeds were used to identify important metabolites, respectively. The rapeseed samples were separated into two clusters of all groups. The results suggest that there were differential metabolites between each pair of countries. The R^2^ and Q^2^ values of the three models were close to 1.0 (Chinese vs. Canadian rapeseeds, R^2^: 0.995, Q^2^: 0.993; Chinese vs. Mongolian rapeseeds, R^2^: 0.953, Q^2^: 0.949; and Canadian vs. Mongolian rapeseeds, R^2^: 0.995, Q^2^: 0.989), suggesting that the models were of good fitness and predictability. The S-plots showed the variable importance in OPLS-DA models and could be used to identify the differential metabolites between every two countries.

### 3.4. Identification of Differential Metabolites of the Three Groups

Univariate analysis methods are the most common methods used for exploratory data analysis. Univariate analysis performed on Metaboanalyst 4.0 can provide a volcano plot that combines FC analysis and *t*-tests together. S-plot and univariate analysis were employed to identify the differential metabolites of the three groups to ensure the accuracy and reliability of the results. The differential metabolites were identified using the criteria that the absolute value of reliability correlation [*p*(corr)] of S-plots was greater than 0.8, the *p*-value calculated using *t*-test was less than 0.001, and the FC was more than twice or less than half.

**Figure 2:**
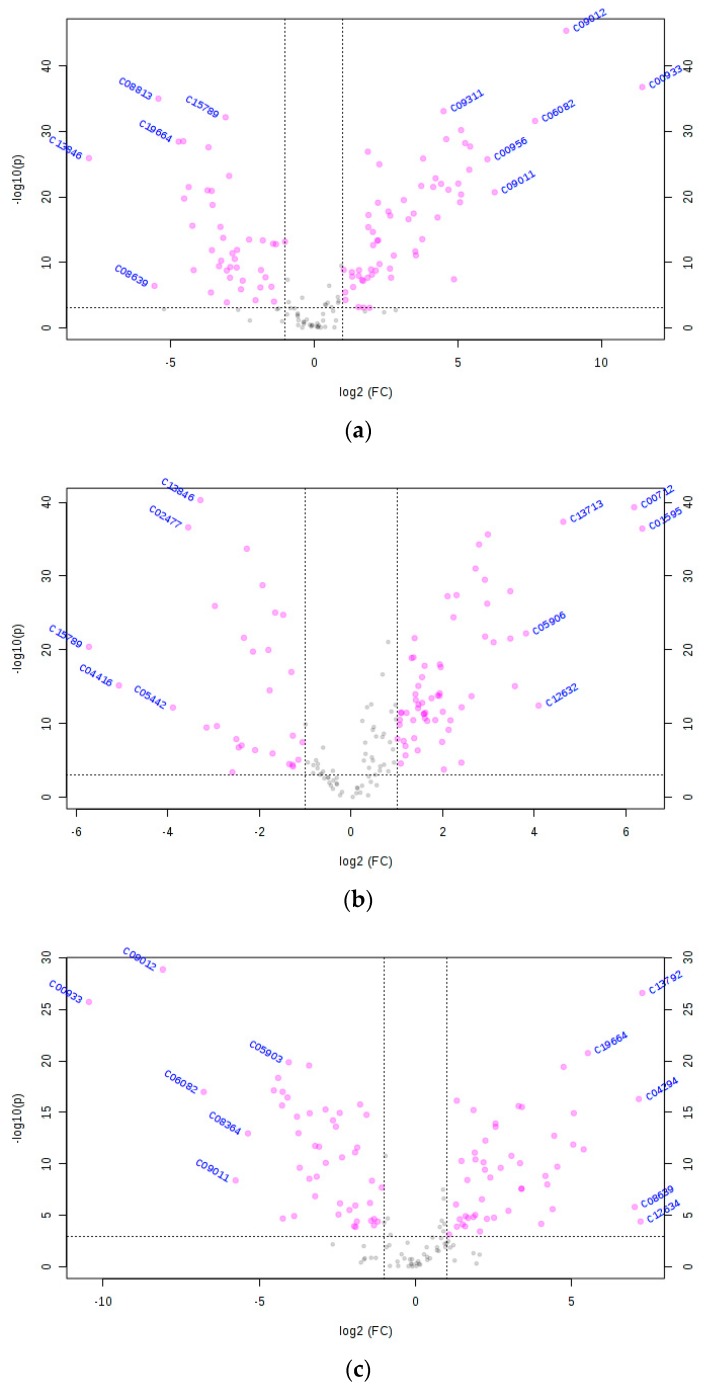
Different metabolites for Chinese and Canadian rapeseeds (**a**), Chinese and Mongolian rapeseeds (**b**), and Canadian and Mongolian rapeseeds (**c**) selected by volcano plot with fold change (FC) threshold (x) 2 and *t*-test threshold (y) 0.001. The FC and *p-*values were both log transformed. The red circles on the left represent metabolites above the thresholds and their contents were up-regulated in Canadian rapeseeds compared with Chinese rapeseeds (**a**), in Mongolian rapeseeds compared with Chinese rapeseeds (**b**), and in Mongolian rapeseeds compared with Canadian rapeseeds (**c**)while the red circles on the right were the opposite. The grey circles represent metabolites with no significant difference. The further its position away from (0,0), the more significant the metabolite was.

Important metabolites with FC threshold (x) 2 and *t*-test threshold (y) 0.001 are displayed by volcano plots in Figure 2. Both FC and *p*-values were log transformed, and the different metabolites are shown as red circles above the threshold.

Based on the results of the S-plots and volcano plots, the comparison between Chinese and Canadian rapeseeds was performed, and 67 metabolites out of 152 were identified as differential metabolites based on the criteria. The 67 metabolites, as listed in Table S2, include phenylpropanoids (12), flavonoids (11), terpenoids (15), alkaloids (2), amino acids (2), amino acid-related compounds (4), fatty acids (11), lipids (2), organic acids (2), polyketides (3), and others. Compared to Canadian rapeseeds, 40 metabolites were up-regulated and 27 metabolites were down-regulated in Chinese rapeseeds. Similarly, 53 out of 152 metabolites—including phenylpropanoids (7), flavonoids (11), terpenoids (7), alkaloids (1), amino acids (4), amino acid-related compounds (6), fatty acids (6), lipids (2), organic acids (3), polyketides (1), and others—were identified as differential metabolites between Chinese and Mongolian rapeseeds (Table S3). Among these metabolites, the contents of 38 metabolites were higher in Chinese rapeseed. Approximately 68 metabolites—including alkaloids (2), amino acids (3), amino acid-related compounds (4), fatty acids (10), flavonoids (19), lipids (3), organic acids (1), phenylpropanoids (13), polyketides (3), terpenoids (7), and others—were significantly different between Canadian and Mongolian rapeseeds (Table S4). Among these metabolites, 32 metabolites were up-regulated in Canadian rapeseeds compared to Mongolian rapeseeds (Table S4).

### 3.5. Metabolic Pathway Analysis of Differential Metabolites Among the Three Groups

**Figure 3:**
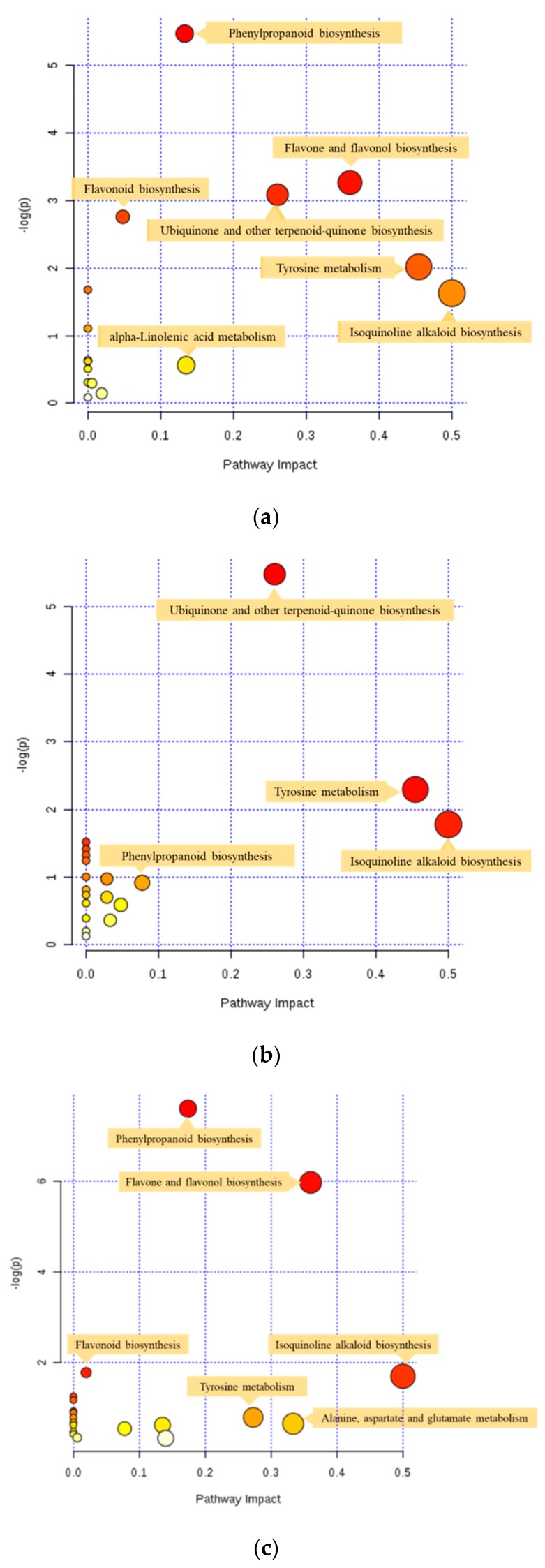
Pathway analyses of differential metabolites between Chinese and Canadian rapeseeds (**a**), Chinese and Mongolian rapeseeds (**b**), and Canadian and Mongolian rapeseeds (**c**). Every circle represents one pathway, and the deeper color represents the more significant changes of the metabolites in the related pathway. Meanwhile, the larger circle means the higher centrality of the metabolite in the related pathways.

The pathway analysis module of MetaboAnalyst 4.0 uses high-quality KEGG metabolic pathways as the back-end knowledge, and combines the results from powerful pathway enrichment analysis with pathway topology analysis to help researchers identify the most relevant pathways involved in the conditions under study. The pathways of the differential metabolites of Chinese vs. Mongolian rapeseeds, Chinese vs. Canadian rapeseeds, and Mongolian vs. Canadian rapeseeds shown in Figure 3 were obtained by the KEGG metabolic pathway database, the hypergeometric test for over representation analysis, and the relative-betweenness centrality for pathway topology analysis. The vertical axis [-log(p)] indicates the significance of the metabolic pathway enrichment, and the horizontal axis indicates the impact of the pathway obtained by pathway topology analysis. The deeper the color, the more significant the changes of the metabolites in the related pathway, and the larger the circle, the higher the centrality of the metabolite in the related pathways [21].

The differential metabolites between Chinese and Canadian rapeseeds were involved in 16 pathways. Based on the characteristics of the bubble plot in Figure 3, phenylpropanoid biosynthesis, flavone and flavonol biosynthesis, and ubiquinone and other terpenoid-quinone biosynthesis were significantly altered in Chinese and Canadian rapeseeds. Similarly, the differential metabolites between Chinese and Mongolian rapeseeds were involved in 19 pathways, whereas the differential metabolites between Canadian and Mongolian rapeseeds were involved in 19 pathways. The metabolic pathways differentially altered between Chinese and Mongolian rapeseeds mainly include ubiquinone and other terpenoid-quinone biosynthesis, tyrosine metabolism, and isoquinoline alkaloid biosynthesis. The metabolic pathways differentially altered between Canadian and Mongolian rapeseeds mainly include phenylpropanoid biosynthesis, flavone and flavonol biosynthesis, and isoquinoline alkaloid biosynthesis. Among the metabolic pathways, phenylpropanoid biosynthesis significantly differed between Chinese and Canadian rapeseeds (*p* < 0.01).

### 3.6. Significantly Differential Metabolites Between Canadian and Chinese Rapeseeds

Canada and China are the biggest producers of rapeseeds, and the rapeseeds of the two countries are the biggest sources of rapeseeds consumed in China. The qualities of Canadian and Chinese rapeseeds attract more and more attention of processing enterprises and consumers.

The metabolic pathways of flavone and flavonol biosynthesis and phenylpropanoid biosynthesis were differentially altered between Chinese and Canadian rapeseeds from the results of metabolic pathway analysis. The metabolites in the two pathways showing significant differences in the two countries include kaempferol, luteolin, coniferyl alcohol, ferulate, coniferin, sinapate, 1-O-sinapoyl-beta-D-glucose, and sinapine. The relative contents of these compounds of rapeseed collected from the two countries are shown in Table S2.

The relative contents of kaempferol, luteolin, coniferin, sinapate, 1-O-sinapoyl-beta-D-glucose, and sinapine of Chinese rapeseeds were significantly higher than those of Canadian rapeseeds (Table S2). Whereas the relative contents of coniferyl alcohol, ferulate, stigmasterol, brassicasterol, and campestanol in Canadian rapeseeds were significantly higher than in Chinese rapeseeds. The contents of metabolites in the downstream of phenylpropanoid biosynthesis in Chinese rapeseeds were higher than in Canadian rapeseeds, according to the pathway of phenylpropanoid biosynthesis in KEGG.

Sinapate, commonly known as sinapic acid, is an important bioactive substance with antioxidant, anti-inflammatory, anticancer, antimutagenic, antiglycemic, neuroprotective, and antibacterial activities [26]. Sinapate is the main free phenolic acid and sinapine, the choline ester of sinapate, is the main phenolic ester in rapeseed. Their amounts are near to 8 g/kg in rapeseed [27,28]. Although sinapate and sinapine occur in rapeseed with higher contents, only a small portion can be transferred into rapeseed oil owing to their hydrophilicity, and most of them remain in the meal, giving a bitter taste and lower digestibility to the meal [29,30]. However, during the process of roasting of rapeseed, canolol (2,6-dimethoxy-4-vinylphenol) can be formed by thermal decarboxylation of sinapate [30]. The antioxidant activity of canolol is equivalent to γ-tocopherol, higher than α-tocopherol and β-carotene, and its antimutagenic potency comparable to ebselen, higher than α-tocopherol and flavonoids [31]. Canolol is lipophilic and not only can it increase the oxidative stability of rapeseed oil, but it can also be used as a dietary supplement to prevent and fight oxidative stress in living organisms [32]. Chinese rapeseeds have a higher content of sinapate, so it is necessary for rapeseed oil-processing enterprises to do some pretreatments to Chinese rapeseeds, such as infrared, microwave roasting, and/or high-temperatures, to enhance the canolol content in rapeseed oil [33]. Thereby, rapeseed oil with a higher content of canolol can be obtained and the nutritional value of rapeseed oil can be improved.

Apart from sinapate, stigmasterol, brassicasterol, and campestanol detected in this study are also well known for their higher contents in rapeseeds. Phytosterols share a similar structure with cholesterol and are structural components of plant membranes. Therefore, they can regulate the physicochemical properties of cell membranes to respond to abiotic and biotic stress [34]. By inhibiting intestinal cholesterol absorption in humans, phytosterols can decrease blood total cholesterol (TC) and low-density lipoprotein cholesterol (LDL-C). Besides, phytosterols also have some other health-promoting effects, such as anti-inflammatory, immunomodulatory, and anticancer effects [35]. Corn oil, rapeseed oil, and wheat germ oil have the highest phytosterol contents and are the main dietary sources of phytosterols for people [34]. Table S2 shows that Canadian rapeseeds have higher contents of stigmasterol, brassicasterol, and campestanol than those in Chinese rapeseeds. The phytosterol content in rapeseeds could be affected by environmental and cultural factors, and nitrogen is one of the most important factors in rapeseed production [36]. The different contents of phytosterols in rapeseeds from the two countries might be due to the genetic variations and growth conditions of the rapeseeds. It has been reported that a dietary phytosterol intake could alter cholesterol metabolism in a dose-dependent manner [37]. It might be possible to use Canadian rapeseeds as materials to produce rapeseed oils with higher phytosterol contents to improve phytosterol intake of the Chinese.

## 4. Conclusions

In this study, UPLC-Q/TOF-MS-based comparative metabolomics combining chemometric methods was employed to study the chemical composition of Chinese, Canadian, and Mongolian rapeseeds. OPLS-DA loading S-plot and univariate analysis were employed to identify the differential metabolites of the rapeseeds collected from three countries. As results, 67, 53, and 68 differential metabolites were identified in Chinese and Canadian rapeseeds, Chinese and Mongolian rapeseeds, and Canadian and Mongolian rapeseeds, respectively. The metabolic pathway analysis results showed that phenylpropanoid biosynthesis, flavone and flavonol biosynthesis, and ubiquinone and other terpenoid-quinone biosynthesis were differentially altered. Chinese rapeseeds have higher contents of sinapate and sinapine, while Canadian rapeseeds possess higher contents of phytosterols. It is necessary for oil-processing enterprises to choose proper rapeseed materials and processing technologies to produce rapeseed oils with higher canolol or phytosterols contents.

## Supplementary Materials

The following are available online at https://www.mdpi.com/2218-1989/9/8/161/s1, Table S1: Metabolites in rapeseed identified based on UPLC-Q-TOF/MS. Table S2: Differential metabolites between Chinese and Canadian rapeseeds (|*p*(corr)|>0.8, *p*-value<0.001, and FC>2 or <0.5)., Table S3: Differential metabolites between Chinese and Mongolian rapeseeds (|*p*(corr)|>0.8, *p*-value<0.001, and FC>2 or <0.5). Table S4: Differential metabolites between Canadian and Mongolian rapeseeds (|*p*(corr)|>0.8, *p*-value<0.001, and FC>2 or <0.5).
