# Supplementary material for: Comparative Metabolomic Analysis of Rapeseeds from Three Countries
Source: Metabolites. 2019 Aug 1;9(8):161. doi: 10.3390/metabo9080161 (PMC6724143; doi:10.3390/metabo9080161)
Supplement: Supplementary file 1 [file metabolites-09-00161-s001.pdf]

metabolites

MDPI

Article

Comparative Metabolomic Analysis of Rapeseeds from Three Countries

Ruinan Yang [1,2,†], Ligang Deng [3,†], Liangxiao Zhang [1,4,5,†], Xiaofeng Yue [1,5], Jin Mao [1,5], Fei Ma [1,5], Xiupin Wang [1,5], Qi Zhang [1,2,6], Wen Zhang [1,5] and Peiwu Li [1,4,5,6,*]

1 Oil Crops Research Institute, Chinese Academy of Agricultural Sciences, Wuhan 430062, China
2 Key Laboratory of Biology and Genetic Improvement of Oil Crops, Ministry of Agriculture and Rural Affairs, Wuhan 430062, China
3 Institute of Agricultural Quality Standards and Testing Technology Research, Shandong Academy of Agricultural Sciences, Jinan 250100, China
4 Laboratory of Quality and Safety Risk Assessment for Oilseed Products (Wuhan), Ministry of Agriculture and Rural Affairs, Wuhan 430062, China
5 Quality Inspection and Test Center for Oilseed Products, Ministry of Agriculture and Rural Affairs, Wuhan 430062, China
6 Key Laboratory of Detection for Mycotoxins, Ministry of Agriculture and Rural Affairs, Wuhan 430062, China
* Correspondence: peiwuli@oilcrops.cn
† These authors contributed equally to this work.

Table S1. Metabolites in rapeseed identified based on UPLC-Q-TOF/MS.

No.	Chemical Compound	Formula	Class	KEGG No.
1	Homogentisate	C8H8O4	Organic acids	C00544
2	Octanoic acid	C8H16O2	Fatty acids	C06423
3	Scopoletin	C10H8O4	Phenylpropanoids	C01752
4	Herniarin	C10H8O3	Phenylpropanoids	C09268
5	1-Acetoxypinoresinol	C22H24O8	Phenylpropanoids	C10544
6	Coniferyl aldehyde	C10H10O3	Phenylpropanoids	C02666
7	Umbelliferone	C9H6O3	Phenylpropanoids	C09315
8	Dehydrocycloguanandin	C18H14O4	Polyketides	C10055
9	Rutin	C27H30O16	Flavonoids	C05625
10	Tulipanin	C27H31O16	Flavonoids	C16315
11	Luteolin 7-O-beta-D-glucoside	C21H20O11	Flavonoids	C03951
12	N-(L-Arginino)succinate	C10H18N4O6	Amino acids	C03406
13	Chlorogenate	C16H18O9	Phenylpropanoids	C00852
14	cis-2-Hydroxycinnamate	C9H8O3	Phenylpropanoids	C05838
15	Naringenin 7-O-beta-D-glucoside	C21H22O10	Flavonoids	C09099
16	L-2-Aminoadipate	C6H11NO4	Amino acids	C00956
17	Nicotinate	C6H5NO2	Alkaloids	C00253
18	L-Glutamate	C5H9NO4	Amino acids	C00025
19	Glutathione disulfide	C20H32N6O12S2	Amino acid related compounds	C00127
20	L-Tyrosine	C9H11NO3	Amino acids	C00082
21	Leucocyanidin	C15H14O7	Flavonoids	C05906
22	L-Cysteine	C3H7NO2S	Amino acids	C00097
23	Coniferin	C16H22O8	Phenylpropanoids	C00761
24	7-Aminomethyl-7-carbaguanine	C7H9N5O	Others	C16675
25	N-Formylmethionine	C6H11NO3S	Amino acids	C03145
26	5-Amino-4-oxopentanoate	C5H9NO3	Organic acids	C00430
27	2,4-Diacetamido-2,4,6-trideoxy-D-mannopyranose	C10H18N2O5	Others	C20424
28	Xanthurenic acid	C10H7NO4	Amino acid related compounds	C02470
29	N-Methylnicotinate	C7H7NO2	Alkaloids	C01004
30	4-Coumarate	C9H8O3	Phenylpropanoids	C00811
31	Desulfoglucotropeolin	C14H19NO6S	Others	C01069
32	Quercetin 3-sophorotrioside	C33H40O22	Flavonoids	C12668
33	Ferulate	C10H10O4	Phenylpropanoids	C01494
34	Cyanidin 3,5,3'-tri-O-glucoside	C33H41O21	Flavonoids	C08629
35	Kaempferol 3-sophorotrioside	C33H40O21	Flavonoids	C12635
36	Lucenin-2	C27H30O16	Flavonoids	C10102
37	Quercitrin	C21H20O11	Flavonoids	C01750

38	Cyanin	C27H31O16	Flavonoids	C08639
39	cis-3,4-Leucopelargonidin	C15H14O6	Flavonoids	C03648
40	Malonylawobanin	C39H39O22	Flavonoids	C08653
41	Anthranilate	C7H7NO2	Organic acids	C00108
42	Quercetin 3-O-beta-D-glucosyl-(1->2)-beta-D-glucoside	C27H30O17	Flavonoids	C12667
43	Quercetin 3-O-glucoside	C21H20O12	Flavonoids	C05623
44	Caffeyl alcohol	C9H10O3	Phenylpropanoids	C12206
45	Cyanidin	C15H11O6	Flavonoids	C05905
46	Kaempferol	C15H10O6	Flavonoids	C05903
47	trans-2-Hydroxycinnamate	C9H8O3	Phenylpropanoids	C01772
48	Bergaptol	C11H6O4	Phenylpropanoids	C00758
49	Lathodoratin	C11H10O4	Polyketides	C09012
50	5-Hydroxyconiferyl alcohol	C10H12O4	Phenylpropanoids	C12205
51	Sinapine	C16H24NO5	Phenylpropanoids	C00933
52	Khellol glucoside	C19H20O10	Polyketides	C09011
53	Coniferyl alcohol	C10H12O3	Phenylpropanoids	C00590
54	(-)-Jasmonic acid	C12H18O3	Fatty acids	C08491
55	Sinensetin	C20H20O7	Flavonoids	C10186
56	N-Acetylaspartylglutamate	C11H16N2O8	Amino acid related compounds	C12270
57	4',5,6,7-Tetramethoxyflavone	C19H18O6	Flavonoids	C14472
58	4-Hydroxyphenylacetate	C8H8O3	Phenylpropanoids	C00642
59	5-(2-Hydroxyethyl)-4-methylthiazole	C6H9NOS	Alkaloids	C04294
60	Odoratol	C17H18O5	Flavonoids	C09807
61	3-O-Methylquercetin	C16H12O7	Flavonoids	C04443
62	Malvidin-3-(p-coumaroyl)-rutinoside-5-glucoside	C44H51O23	Flavonoids	C16296
63	Cyanidin 3-O-sophoroside	C27H31O16	Flavonoids	C16306
64	Kaempferol 3-O-beta-D-glucosyl-(1->2)-beta-D-glucoside	C27H30O16	Flavonoids	C12634
65	1-O-Sinapoyl-beta-D-glucose	C17H22O10	Phenylpropanoids	C01175
66	Luteolin	C15H10O6	Flavonoids	C01514
67	Isoswertisin 2''-rhamnoside	C28H32O14	Flavonoids	C12629
68	4-Coumaroylshikimate	C16H16O7	Phenylpropanoids	C02947
69	Quinate	C7H12O6	Organic acids	C00296
70	3-(4-Hydroxyphenyl)lactate	C9H10O4	Others	C03672
71	Sinapate	C11H12O5	Phenylpropanoids	C00482
72	1'-Acetoxychavicol acetate	C13H14O4	Phenylpropanoids	C10426
73	Tangeretin	C20H20O7	Flavonoids	C10190
74	Samaderin A	C18H18O6	Terpenoids	C08781
75	Abscisic acid glucose ester	C21H30O9	Others	C15970
76	7,8-Dihydroxycoumarin	C9H6O4	Phenylpropanoids	C03093
77	Goyazensolide	C19H20O7	Terpenoids	C09467
78	Eucommin A	C27H34O12	Phenylpropanoids	C10560

79	Catechin 7-O-beta-D-xyloside	C20H22O10	Flavonoids	C09617
80	Isorhamnetin	C16H12O7	Flavonoids	C10084
81	Iridin	C24H26O13	Flavonoids	C10465
82	Alectrol	C21H24O7	Terpenoids	C09059
83	2-Methoxyestrone 3-sulfate	C19H24O6S	Terpenoids	C08358
84	Cyanidin 3-O-(6''-glucosyl-2''-xylosylgalactoside)	C32H39O20	Flavonoids	C08612
85	Fraxetin	C10H8O5	Phenylpropanoids	C09265
86	4,8-Dihydroxyquinoline	C9H7NO2	Amino acid related compounds	C05637
87	Swietenidin B	C11H11NO3	Alkaloids	C10741
88	Dalpanin	C26H30O12	Flavonoids	C10416
89	Abscisate	C15H20O4	Terpenoids	C06082
90	Aurantio-obtusin beta-D-glucoside	C23H24O12	Polyketides	C10303
91	Poncirin	C28H34O14	Flavonoids	C09830
92	Luteolin 7-O-[beta-D-glucuronosyl-(1->2)-beta-D-glucuronide]	C27H26O18	Flavonoids	C12632
93	Anthemis glycoside A	C39H49NO21	Amino acid related compounds	C08326
94	7-Methylxanthosine	C11H15N4O6	Alkaloids	C16352
95	Vicianin	C19H25NO10	Amino acid related compounds	C01870
96	Ononin	C22H22O9	Flavonoids	C10509
97	2-Oxo-10-methylthiodecanoic acid	C11H20O3S	Fatty acids	C17232
98	3,7,4'-Tri-O-methylquercetin	C18H16O7	Flavonoids	C04444
99	5-(2'-Carboxyethyl)-4,6-dihydroxypicolinate	C9H9NO6	Amino acid related compounds	C05655
100	Delphinidin 5-O-beta-D-glucoside 3-O-beta-D-sambubioside	C32H39O21	Others	C20494
101	Coumarin	C9H6O2	Phenylpropanoids	C05851
102	Scoparone	C11H10O4	Phenylpropanoids	C09311
103	Fruticosonine	C20H28N2O	Alkaloids	C09191
104	(S)-Norlaudanosoline	C16H17NO4	Alkaloids	C02916
105	2-Oxo-7-methylthioheptanoic acid	C8H14O3S	Fatty acids	C17220
106	Pimelic acid	C7H12O4	Fatty acids	C02656
107	Hexadecasphinganine	C16H35NO2	Lipids	C13915
108	(15Z)-12-Oxophyto-10,15-dienoic acid	C18H28O3	Fatty acids	C01226
109	Helminthosporol	C15H24O2	Terpenoids	C09678
110	(9Z,11E)-(13S)-13-Hydroperoxyoctadeca-9,11-dienoic acid	C18H32O4	Fatty acids	C04717
111	(9Z)-(13S)-12,13-Epoxyoctadeca-9,11-dienoic acid	C18H30O3	Fatty acids	C04594
112	2-Oxooctadecanoic acid	C18H34O3	Fatty acids	C00869
113	Phytosphingosine	C18H39NO3	Lipids	C12144
114	Stearidonic acid	C18H28O2	Fatty acids	C16300
115	5alpha-Ergosta-7,22-diene-3beta,5-diol	C28H46O2	Terpenoids	C04416
116	Cucurbitacin D	C30H44O7	Terpenoids	C08796
117	2-Methoxy-5Z-hexadecenoic acid	C17H32O3	Fatty acids	C13792
118	Allotetrahydrodeoxycorticosterone	C21H34O3	Others	C13713
119	Proanthocyanidin A2	C30H24O12	Flavonoids	C10237

120	(6Z,9Z,12Z)-Octadecatrienoic acid	C18H30O2	Fatty acids	C06426
121	Mukaadial	C15H22O4	Terpenoids	C09702
122	2-trans,6-trans-Farnesal	C15H24O	Terpenoids	C03461
123	Octadecanamide	C18H37NO	Lipids	C13846
124	Ricinoleic acid	C18H34O3	Fatty acids	C08365
125	(9Z,12Z)-(8R)-Hydroxyoctadeca-9,12-dienoic acid	C18H32O3	Fatty acids	C08318
126	Capsidiol	C15H24O2	Terpenoids	C09627
127	Brassicasterol	C28H46O	Terpenoids	C08813
128	(11E)-Octadecenoic acid	C18H34O2	Fatty acids	C08367
129	Campestanol	C28H50O	Terpenoids	C15787
130	Geranylgeraniol	C20H34O	Terpenoids	C09094
131	Stigmasterol	C29H48O	Terpenoids	C05442
132	Malvalic acid	C18H32O2	Fatty acids	C08321
133	Vernolic acid	C18H32O3	Fatty acids	C08368
134	2,7,11-Cembratrien-4,6-diol	C20H34O2	Terpenoids	C09072
135	Punicic acid	C18H30O2	Fatty acids	C08364
136	Sterculic acid	C19H34O2	Fatty acids	C08366
137	(9Z)-Hexadecenoic acid	C16H30O2	Fatty acids	C08362
138	Sterol 3-beta-D-glucoside	C23H38O6	Terpenoids	C03641
139	Phylloquinol	C31H48O2	Others	C03313
140	Ergostane	C28H50	Terpenoids	C19664
141	Linoleic acid	C18H32O2	Fatty acids	C01595
142	p-Coumaroylagmatine	C14H20N4O2	Amino acid related compounds	C04498
143	Casbene	C20H32	Terpenoids	C01414
144	2-Phytyl-1,4-naphthoquinone	C30H44O2	Terpenoids	C13309
145	(9Z)-Octadecenoic acid	C18H34O2	Fatty acids	C00712
146	(24R,24(1)R)-Fucosterol epoxide	C29H48O2	Terpenoids	C03910
147	alpha-Tocopherol	C29H50O2	Others	C02477
148	Betulin	C30H50O2	Terpenoids	C08618
149	Cathasterone	C28H48O3	Terpenoids	C15790
150	6-Oxocampestanol	C28H48O2	Terpenoids	C15789
151	alpha-Oxo-benzeneacetic acid	C8H6O3	Organic acids	C02137
152	Gallate	C7H6O5	Organic acids	C01424

Table S2. Differential metabolites between Chinese and Canadian rapeseeds (|*p*(corr)|>0.8, *p*-value<0.001, and FC>2 or <0.5).

No.	FC	p value	|*p*(corr)|	Chemical Compound	Formula	Class
1	437.04	4.04E-46	0.99621	Lathodoratin	C11H10O4	Polyketides
2	2732.8	1.62E-37	0.99251	Sinapine	C16H24NO5	Phenylpropanoids
3	0.023474	9.72E-36	0.99187	Brassicasterol	C28H46O	Terpenoids
4	0.11855	6.82E-33	0.98934	6-Oxocampestanol	C28H48O2	Terpenoids
5	22.726	7.92E-34	0.98848	Scoparone	C11H10O4	Phenylpropanoids
6	206.32	2.47E-32	0.98588	Abscisate	C15H20O4	Terpenoids
7	34.68	6.20E-31	0.9851	Lucenin-2	C27H30O16	Flavonoids
8	0.042901	3.15E-29	0.98292	2-Methoxy-5Z-hexadecenoic acid	C17H32O3	Fatty acids
9	38.225	6.05E-29	0.98276	Luteolin	C15H10O6	Flavonoids
10	0.038253	3.58E-29	0.9823	Ergostane	C28H50	Terpenoids
11	24.262	1.51E-29	0.98206	Kaempferol	C15H10O6	Flavonoids
12	43.065	1.99E-28	0.97958	Allotetrahydrodeoxycorticosterone	C21H34O3	Others
13	0.07857	2.63E-28	0.97896	Campestanol	C28H50O	Terpenoids
14	3.6625	1.24E-27	0.97713	Coniferin	C16H22O8	Phenylpropanoids
15	13.882	1.38E-26	0.97535	Vernolic acid	C18H32O3	Fatty acids
16	0.0044011	1.21E-26	0.97503	Octadecanamide	C18H37NO	Lipids
17	4.829	1.04E-25	0.97438	Sterol 3-beta-D-glucoside	C23H38O6	Terpenoids
18	65.33	1.79E-26	0.97434	L-2-Aminoadipate	C6H11NO4	Amino acids
19	42.202	7.01E-25	0.9711	(9Z)-Octadecenoic acid	C18H34O2	Fatty acids
20	0.12919	6.27E-24	0.96828	Capsidiol	C15H24O2	Terpenoids
21	18.757	1.43E-23	0.96495	Linoleic acid	C18H32O2	Fatty acids
22	0.048788	3.17E-22	0.96104	Stigmasterol	C29H48O	Terpenoids
23	13.267	2.09E-22	0.96067	Proanthocyanidin A2	C30H24O12	Flavonoids
24	17.715	3.04E-22	0.96061	Catechin 7-O-beta-D-xyloside	C20H22O10	Flavonoids
25	21.412	1.03E-22	0.9596	Abscisic acid glucose ester	C21H30O9	Others
26	32.388	9.19E-23	0.95929	Kaempferol 3-sophorotrioside	C33H40O21	Flavonoids
27	0.076706	1.00E-21	0.95659	Gallate	C7H6O5	Organic acids
28	0.084899	1.24E-21	0.95491	3-(4-Hydroxyphenyl)lactate	C9H10O4	Others
29	77.905	1.97E-21	0.95469	Khellol glucoside	C19H20O10	Polyketides
30	34.685	4.21E-21	0.95284	Quercitrin	C21H20O11	Flavonoids
31	25.544	8.28E-22	0.9522	L-Tyrosine	C9H11NO3	Amino acids
32	8.7044	3.10E-20	0.94661	Samaderin A	C18H18O6	Terpenoids
33	0.043726	1.80E-20	0.94647	5alpha-Ergosta-7,22-diene-3beta,5-diol	C28H46O2	Terpenoids
34	4.6732	7.84E-20	0.94375	Cyanidin	C15H11O6	Flavonoids
35	33.801	6.62E-20	0.94316	Dalpanin	C26H30O12	Flavonoids
36	0.086672	1.73E-19	0.94236	Xanthurenic acid	C10H7NO4	Amino acid related compounds
37	6.0087	1.72E-18	0.9321	Phytosphingosine	C18H39NO3	Lipids

38	11.047	3.54E-18	0.93024	Cyanidin 3,5,3'-tri-O-glucoside	C33H41O21	Flavonoids
39	19.677	1.40E-17	0.92853	Punicic acid	C18H30O2	Fatty acids
40	6.2822	7.48E-18	0.92627	(9Z)-Hexadecenoic acid	C16H30O2	Fatty acids
41	3.7185	6.00E-18	0.92605	p-Coumaroylagmatine	C14H20N4O2	Amino acid related compounds
42	9.7523	2.64E-17	0.91969	cis-3,4-Leucopelargonidin	C15H14O6	Flavonoids
43	0.053333	2.59E-16	0.91312	5-(2-Hydroxyethyl)-4-methylthiazole	C6H9NOS	Alkaloids
44	3.7067	4.28E-16	0.91152	Coumarin	C9H6O2	Phenylpropanoids
45	0.1048	3.86E-16	0.9063	Goyazensolide	C19H20O7	Terpenoids
46	4.1359	2.33E-15	0.90201	2-Phytyl-1,4-naphthoquinone	C30H44O2	Terpenoids
47	0.1119	1.86E-14	0.89284	Homogentisate	C8H8O4	Organic acids
48	0.20936	3.40E-14	0.88853	Ferulate	C10H10O4	Phenylpropanoids
49	4.5995	5.08E-14	0.8872	Sinapate	C11H12O5	Phenylpropanoids
50	0.49741	7.09E-14	0.8839	trans-2-Hydroxycinnamate	C9H8O3	Phenylpropanoids
51	4.694	4.30E-14	0.88334	Vicianin	C19H25NO10	Amino acid related compounds
52	0.29132	4.52E-14	0.88283	Herniarin	C10H8O3	Phenylpropanoids
53	13.606	2.94E-14	0.87842	Stearidonic acid	C18H28O2	Fatty acids
54	0.4003	1.70E-13	0.87738	Umbelliferone	C9H6O3	Phenylpropanoids
55	4.1622	2.41E-13	0.87602	1-O-Sinapoyl-beta-D-glucose	C17H22O10	Phenylpropanoids
56	0.37351	1.46E-13	0.87589	Coniferyl alcohol	C10H12O3	Phenylpropanoids
57	0.08541	1.42E-12	0.86574	Betulin	C30H50O2	Terpenoids
58	0.15651	1.29E-12	0.85973	1-Acetoxypinoresinol	C22H24O8	Phenylpropanoids
59	0.13966	4.24E-12	0.85116	7-Methylxanthosine	C11H15N4O6	Alkaloids
60	11.499	2.16E-12	0.84759	(6Z,9Z,12Z)-Octadecatrienoic acid	C18H30O2	Fatty acids
61	6.8409	9.24E-12	0.84744	5-(2'-Carboxyethyl)-4,6-dihydroxypicolinate	C9H9NO6	Amino acid related compounds
62	11.676	8.69E-12	0.84386	Dehydrocycloguanandin	C18H14O4	Polyketides
63	0.14803	3.07E-11	0.83715	Helminthosporol	C15H24O2	Terpenoids
64	0.10659	5.70E-11	0.8315	Pimelic acid	C7H12O4	Fatty acids
65	4.8487	1.87E-10	0.81461	2-Oxo-7-methylthioheptanoic acid	C8H14O3S	Fatty acids
66	0.13294	5.32E-10	0.80682	(15Z)-12-Oxophyto-10,15-dienoic acid	C18H28O3	Fatty acids
67	0.10135	3.95E-10	0.80368	(24R,24(1)R)-Fucosterol epoxide	C29H48O2	Terpenoids

Table S3. Differential metabolites between Chinese and Mongolian rapeseeds (|*p*(corr)|>0.8, *p*-value<0.001, and FC>2 or <0.5).

No.	FC	p value	|*p*(corr)|	Chemical Compound	Formula	Class
1	0.10235	4.89E-41	0.98288	Octadecanamide	C18H37NO	Lipids
2	72.475	4.45E-40	0.98126	(9Z)-Octadecenoic acid	C18H34O2	Fatty acids
3	7.9253	2.23E-36	0.98034	alpha-Oxo-benzeneacetic acid	C8H6O3	Organic acids
4	24.778	4.22E-38	0.97814	Allotetrahydrodeoxycorticosterone	C21H34O3	Others
5	6.9315	5.07E-35	0.97672	2-Phytyl-1,4-naphthoquinone	C30H44O2	Terpenoids
6	0.20634	1.95E-34	0.97612	Umbelliferone	C9H6O3	Phenylpropanoids
7	81.921	3.58E-37	0.97581	Linoleic acid	C18H32O2	Fatty acids
8	0.085075	2.43E-37	0.97494	alpha-Tocopherol	C29H50O2	Others
9	6.5668	9.52E-32	0.96813	2-Methoxy-5Z-hexadecenoic acid	C17H32O3	Fatty acids
10	7.5693	3.19E-30	0.96562	5-(2-Hydroxyethyl)-4-methylthiazole	C6H9NOS	Alkaloids
11	0.26203	1.77E-29	0.96227	Scopoletin	C10H8O4	Phenylpropanoids
12	4.9317	3.90E-28	0.96048	Malvidin-3-(p-coumaroyl)-rutinoside-5-glucoside	C44H51O23	Flavonoids
13	4.3068	5.43E-28	0.95908	Phylloquinol	C31H48O2	Others
14	11.128	1.15E-28	0.95369	(9Z)-Hexadecenoic acid	C16H30O2	Fatty acids
15	0.1274	1.16E-26	0.95146	Coniferyl aldehyde	C10H10O3	Phenylpropanoids
16	7.8436	5.58E-27	0.95037	p-Coumaroylagmatine	C14H20N4O2	Amino acid related compounds
17	0.31712	9.20E-26	0.94679	Herniarin	C10H8O3	Phenylpropanoids
18	4.7102	3.95E-25	0.94186	N-Acetylaspartylglutamate	C11H16N2O8	Amino acid related compounds
19	0.35819	1.87E-25	0.93469	Gallate	C7H6O5	Organic acids
20	7.5966	1.63E-22	0.93442	5-(2'-Carboxyethyl)-4,6-dihydroxypicolinate	C9H9NO6	Amino acid related compounds
21	14.111	6.12E-23	0.92447	Leucocyanidin	C15H14O7	Flavonoids
22	2.6142	2.70E-22	0.92257	Sterol 3-beta-D-glucoside	C23H38O6	Terpenoids
23	0.19755	2.44E-22	0.92174	Brassicasterol	C28H46O	Terpenoids
24	11.134	3.02E-22	0.916	Glutathione disulfide	C20H32N6O12S2	Amino acid related compounds
25	8.638	9.66E-22	0.91377	Chlorogenate	C16H18O9	Phenylpropanoids
26	0.018926	4.06E-21	0.911	6-Oxocampestanol	C28H48O2	Terpenoids
27	2.5696	1.10E-19	0.91096	N-Formylmethionine	C6H11NO3S	Amino acids
28	2.4978	1.36E-19	0.90883	Ricinoleic acid	C18H34O3	Fatty acids
29	0.22664	1.81E-20	0.90352	Cucurbitacin D	C30H44O7	Terpenoids
30	0.28636	1.07E-20	0.90252	Phytosphingosine	C18H39NO3	Lipids
31	3.8826	2.30E-18	0.89758	Quercitrin	C21H20O11	Flavonoids
32	3.0421	1.56E-18	0.89174	Aurantio-obtusin beta-D-glucoside	C23H24O12	Polyketides
33	3.8297	9.92E-19	0.89137	L-2-Aminoadipate	C6H11NO4	Amino acids
34	2.9276	5.36E-17	0.88393	Malonylawobanin	C39H39O22	Flavonoids
35	0.40502	1.08E-17	0.87671	Homogentisate	C8H8O4	Organic acids
36	2.7657	8.44E-16	0.8593	Cyanin	C27H31O16	Flavonoids
37	0.029809	7.17E-16	0.8583	5alpha-Ergosta-7,22-diene-3beta,5-diol	C28H46O2	Terpenoids

38	0.29164	3.26E-15	0.85223	1-Acetoxypinoresinol	C22H24O8	Phenylpropanoids
39	11.928	8.93E-16	0.852	N-(L-Arginino)succinate	C10H18N4O6	Amino acids
40	2.6464	1.12E-14	0.84727	Iridin	C24H26O13	Flavonoids
41	3.8109	1.87E-14	0.84092	7,8-Dihydroxycoumarin	C9H6O4	Phenylpropanoids
42	3.8244	8.24E-15	0.83541	2-Oxo-10-methylthiodecanoic acid	C11H20O3S	Fatty acids
43	3.3796	3.88E-14	0.8332	Isorhamnetin	C16H12O7	Flavonoids
44	3.7093	1.67E-14	0.83045	Naringenin 7-O-beta-D-glucoside	C21H22O10	Flavonoids
45	2.6622	6.83E-14	0.83045	Lucenin-2	C27H30O16	Flavonoids
46	6.1863	2.05E-14	0.82635	Delphinidin 5-O-beta-D-glucoside 3-O-beta-D-sambubioside	C32H39O21	Others
47	2.9322	1.68E-13	0.81983	Abscisic acid glucose ester	C21H30O9	Others
48	5.3292	6.52E-13	0.81506	3-O-Methylquercetin	C16H12O7	Flavonoids
49	2.7713	2.85E-13	0.81414	Anthemis glycoside A	C39H49NO21	Amino acid related compounds
50	0.067521	7.11E-13	0.81346	Stigmasterol	C29H48O	Terpenoids
51	17.083	3.83E-13	0.8061	Luteolin 7-O-[beta-D-glucuronosyl-(1->2)-beta-D-glucuronide]	C27H26O18	Flavonoids
52	2.3141	3.77E-12	0.80361	Xanthurenic acid	C10H7NO4	Amino acid related compounds
53	2.7586	8.65E-13	0.8023	L-Tyrosine	C9H11NO3	Amino acids

Table S4. Differential metabolites between Canadian and Mongolian rapeseeds (|*p*(corr)|>0.8, *p*-value<0.001, and FC>2 or <0.5).

No.	FC	p value	|*p*(corr)|	Chemical Compound	Formula	Class
1	0.003686	1.36E-29	0.99673	Lathodoratin	C11H10O4	Polyketides
2	153.07	2.50E-27	0.99569	2-Methoxy-5Z-hexadecenoic acid	C17H32O3	Fatty acids
3	0.000716	1.86E-26	0.99424	Sinapine	C16H24NO5	Phenylpropanoids
4	45.817	1.73E-21	0.98976	Ergostane	C28H50	Terpenoids
5	0.060205	1.34E-20	0.98815	Kaempferol	C15H10O6	Flavonoids
6	0.094489	2.75E-20	0.98499	Vernolic acid	C18H32O3	Fatty acids
7	26.7	3.77E-20	0.98465	Xanthurenic acid	C10H7NO4	Amino acid related compounds
8	0.047658	4.47E-19	0.98121	Phytosphingosine	C18H39NO3	Lipids
9	0.009135	1.01E-17	0.97774	Abscisate	C15H20O4	Terpenoids
10	0.058621	3.51E-17	0.97763	L-2-Aminoadipate	C6H11NO4	Amino acids
11	141.93	4.98E-17	0.97602	5-(2-Hydroxyethyl)-4-methylthiazole	C6H9NOS	Alkaloids
12	0.043243	7.28E-18	0.97465	Stearidonic acid	C18H28O2	Fatty acids
13	0.29311	1.69E-16	0.9746	Cyanidin	C15H11O6	Flavonoids
14	0.052581	1.00E-17	0.9745	(6Z,9Z,12Z)-Octadecatrienoic acid	C18H30O2	Fatty acids
15	0.18656	1.13E-15	0.9732	Luteolin 7-O-beta-D-glucoside	C21H20O11	Flavonoids
16	2.5022	7.21E-17	0.97304	trans-2-Hydroxycinnamate	C9H8O3	Phenylpropanoids
17	9.8622	2.39E-16	0.97297	Capsidiol	C15H24O2	Terpenoids
18	0.051945	2.11E-16	0.97203	Dalpanin	C26H30O12	Flavonoids
19	33.542	1.18E-15	0.96731	Pimelic acid	C7H12O4	Fatty acids
20	10.626	2.97E-16	0.96688	7,8-Dihydroxycoumarin	C9H6O4	Phenylpropanoids
21	0.13604	5.17E-16	0.96664	Coniferyl aldehyde	C10H10O3	Phenylpropanoids
22	0.33802	1.70E-15	0.96632	Coniferin	C16H22O8	Phenylpropanoids
23	3.6236	5.90E-16	0.96604	Ferulate	C10H10O4	Phenylpropanoids
24	0.072263	2.61E-15	0.96477	Scoparone	C11H10O4	Phenylpropanoids
25	0.095785	1.20E-15	0.96399	2-Methoxyestrone 3-sulfate	C19H24O6S	Terpenoids
26	0.16067	5.81E-15	0.96262	Proanthocyanidin A2	C30H24O12	Flavonoids
27	5.9056	1.19E-14	0.9551	L-Glutamate	C5H9NO4	Amino acids
28	5.9162	2.50E-14	0.9543	3-(4-Hydroxyphenyl)lactate	C9H10O4	Others
29	0.024365	1.08E-13	0.95357	Punicic acid	C18H30O2	Fatty acids
30	0.17128	2.40E-14	0.95324	Vicianin	C19H25NO10	Amino acid related compounds
31	21.72	1.85E-13	0.94825	Goyazensolide	C19H20O7	Terpenoids
32	0.074695	1.04E-13	0.94507	Dehydrocycloguanandin	C18H14O4	Polyketides
33	4.7112	5.46E-13	0.94379	Sinensetin	C20H20O7	Flavonoids
34	33.134	1.32E-12	0.94217	Cyanidin 3-O-(6''-glucosyl-2''-xylosylgalactoside)	C32H39O20	Flavonoids
35	0.27533	2.52E-12	0.94112	Tulipanin	C27H31O16	Flavonoids
36	0.11802	2.14E-12	0.93485	Catechin 7-O-beta-D-xyloside	C20H22O10	Flavonoids
37	0.10799	1.76E-12	0.93352	L-Tyrosine	C9H11NO3	Amino acids

38	41.764	3.89E-12	0.9306	Luteolin 7-O-[beta-D-glucuronosyl-(1->2)-beta-D-glucuronide]	C27H26O18	Flavonoids
39	8.4155	1.65E-11	0.93057	Brassicasterol	C28H46O	Terpenoids
40	0.26219	7.57E-12	0.92721	Hexadecasphinganine	C16H35NO2	Lipids
41	3.7259	8.04E-12	0.92442	Coniferyl alcohol	C10H12O3	Phenylpropanoids
42	2.7754	5.17E-11	0.92057	Malvidin-3-(p-coumaroyl)-rutinoside-5-glucoside	C44H51O23	Flavonoids
43	0.19645	2.31E-11	0.91992	Scopoletin	C10H8O4	Phenylpropanoids
44	3.7826	3.70E-11	0.91911	N-Acetylaspartylglutamate	C11H16N2O8	Amino acid related compounds
45	0.13694	7.92E-11	0.9126	Abscisic acid glucose ester	C21H30O9	Others
46	4.5251	6.99E-11	0.91179	(15Z)-12-Oxophyto-10,15-dienoic acid	C18H28O3	Fatty acids
47	10.187	8.54E-11	0.90923	3-O-Methylquercetin	C16H12O7	Flavonoids
48	6.6293	2.41E-10	0.90835	Sterculic acid	C19H34O2	Fatty acids
49	0.076764	2.37E-10	0.90823	Lucenin-2	C27H30O16	Flavonoids
50	23.256	1.87E-10	0.90559	Octadecanamide	C18H37NO	Lipids
51	4.6696	3.70E-10	0.90393	Gallate	C7H6O5	Organic acids
52	0.11194	1.72E-09	0.89191	Quercitrin	C21H20O11	Flavonoids
53	5.2576	2.10E-09	0.88487	Delphinidin 5-O-beta-D-glucoside 3-O-beta-D-sambubioside	C32H39O21	Others
54	17.867	1.47E-09	0.88472	Anthemis glycoside A	C39H49NO21	Amino acid related compounds
55	0.018511	4.03E-09	0.88207	Khellol glucoside	C19H20O10	Polyketides
56	0.095105	2.78E-09	0.88201	Kaempferol 3-sophorotrioside	C33H40O21	Flavonoids
57	0.38154	4.38E-09	0.88169	Rutin	C27H30O16	Flavonoids
58	3.1579	3.68E-09	0.87232	5-Hydroxyconiferyl alcohol	C10H12O4	Phenylpropanoids
59	18.62	9.93E-09	0.86896	Chlorogenate	C16H18O9	Phenylpropanoids
60	0.47353	1.98E-08	0.86484	1-O-Sinapoyl-beta-D-glucose	C17H22O10	Phenylpropanoids
61	10.496	2.61E-08	0.85931	Casbene	C20H32	Terpenoids
62	10.545	2.54E-08	0.85609	7-Methylxanthosine	C11H15N4O6	Alkaloids
63	4.3675	2.73E-07	0.83479	Linoleic acid	C18H32O2	Fatty acids
64	0.10808	1.38E-07	0.82678	(9Z)-(13S)-12,13-Epoxyoctadeca-9,11-dienoic acid	C18H30O3	Fatty acids
65	0.18784	6.80E-07	0.80773	Tangeretin	C20H20O7	Flavonoids
66	2.4598	8.69E-07	0.80476	4-Hydroxyphenylacetate	C8H8O3	Phenylpropanoids
67	0.26396	1.10E-06	0.80259	Ononin	C22H22O9	Flavonoids
68	0.36498	6.28E-07	0.80165	4',5,6,7-Tetramethoxyflavone	C19H18O6	Flavonoids
